# The successful management for long-term intractable enteroatmospheric fistula: A case report

**DOI:** 10.1016/j.amsu.2020.07.044

**Published:** 2020-07-30

**Authors:** Kyota Tatsuta, Takeshi Oshima, Hisato Ishimatsu, Hiroyuki Hazama, Ko Ohata

**Affiliations:** Department of Gastroenterological Surgery, Shizuoka General Hospital, Aoi-ku, Japan

**Keywords:** Enteroatmospheric fistula, Open abdomen management, Negative pressure wound therapy, Case report, EAF, Enteroatmospheric fistula, ECF, Enterocutaneous fistulas, NPWT, Negative pressure wound therapy, OAM, Open abdomen management

## Abstract

**Introduction:**

Efficacy of open abdomen management with negative pressure wound therapy for enteroatmospheric fistula has been performed. But, few reports have shown its utility for enteroatmospheric fistula several years after onset.

**Presentation of case:**

A 46 year-old woman underwent total colectomy due to total ulcerative colitis in her twenties. Three years before the onset of enteroatmospheric fistula, she underwent simple total hysterectomy for uterine smooth muscle tumor. Small bowel obstruction occurred early and a small bowel bypass was performed. However, she had sudden abdominal pain and was diagnosed with anastomotic leakage of small bowel bypass. Although antibiotic treatment was initiated, infection was difficult to control, and a midline abdominal incision was performed, followed by the formation of enteroatmospheric fistula. She declined early surgical intervention and started receiving home parenteral nutrition with antibiotic treatment. Although central vein management was continued, catheter infection became frequent. Hence, surgical intervention was planned 30 months after the formation of enteroatmospheric fistula. Two-stage abdominal wall reconstruction using open abdomen management with negative pressure wound therapy was planned. The definitive abdominal wall reconstruction was performed 14 days after the initial operation. Finally, she was discharged without reoperation.

**Discussion:**

Enteroatmospheric fistula has no overlying soft tissue and no real fistula tract. Besides these complications, there were complications of the scarred abdominal wall from intestinal fluid exposure for 30 months.

**Conclusion:**

The strategy using open abdomen management with negative pressure wound therapy for long-term enteroatmospheric fistula will have a good postoperative outcome with the same as early intervention.

## Highlights

• Negative pressure wound therapy is effective in postonset enteroatmospheric fistula.
• It repairs abdominal wall skin damage from 30-month intestinal fluid exposure.
• Open abdominal management was able to control infection in the perioperative period.

## 1 Introduction

Enteroatmospheric fistula (EAF), which is a subset of enterocutaneous fistulas (ECF) that occur in the setting of an open abdomen, is difficult to treat. This complication occurs in 25% of patients with a reported mortality rate of 42%.^1^ Prevention of EAF is the best treatment strategy but is often the surgical strategy.^2^ Relating to surgical cases, a vast majority of EAF do not close spontaneously; thus, a definitive surgical repair is usually required.

Despite having undergone the definitive surgery, EAF often recurs and may require reoperation because of infection complications.[3], [4], [5] For solution of these problems, previous reports have shown that the efficacy of Open abdomen management (OAM) with negative pressure wound therapy (NPWT) for EAF during the period until the definitive surgery has been performed.^6^ The continuous negative pressure by NPWT facilitates the removal of intestinal fluid and protects the surrounding skin.^7^ As a result, there have been many reports of early intervention using OAM with NPWT for EAF^6^^,^^7^; however, few reports have shown its utility for treatment of EAF several years after the onset.

In this case study, we report the successful definitive management for long-term intractable EAF using OAM with NPWT.

## 2 Presentation of Case

A 46 year-old woman was diagnosed with total ulcerative colitis and underwent total colectomy and ileostomy in her twenties. Three years before the onset of EAF, she underwent simple total hysterectomy for uterine smooth muscle tumor; however, small bowel obstruction occurred three days after the operation. We administered conservative treatment with long-tube insertion because of her strong desire. Her symptoms did not respond to conservative treatment for 1 month, and laparotomy was planned to release small bowel obstruction. Because of intraabdominal adhesion, a small bowel bypass was performed. On the third postoperative day, she had sudden abdominal pain and was diagnosed with anastomotic leakage of small bowel bypass. Antibiotic treatment was started. The infection was difficult to control, and a midline abdominal incision opened up, followed by the formation of EAF. Because EAF was formed from the upper jejunum, there was not enough of her small intestine remaining to absorb sufficient nutrition. She declined early surgical intervention for EAF. Hence, she started receiving home parenteral nutrition with antibiotic treatment for EAF. Although central vein management was continued for 30 months, catheter infection became frequent because of a poor skin environment of atopic dermatitis. Repeated infections had significantly reduced her quality of life. Surgical intervention for EAF was planned 30 months after the formation of EAF.

**Fig. 1:**
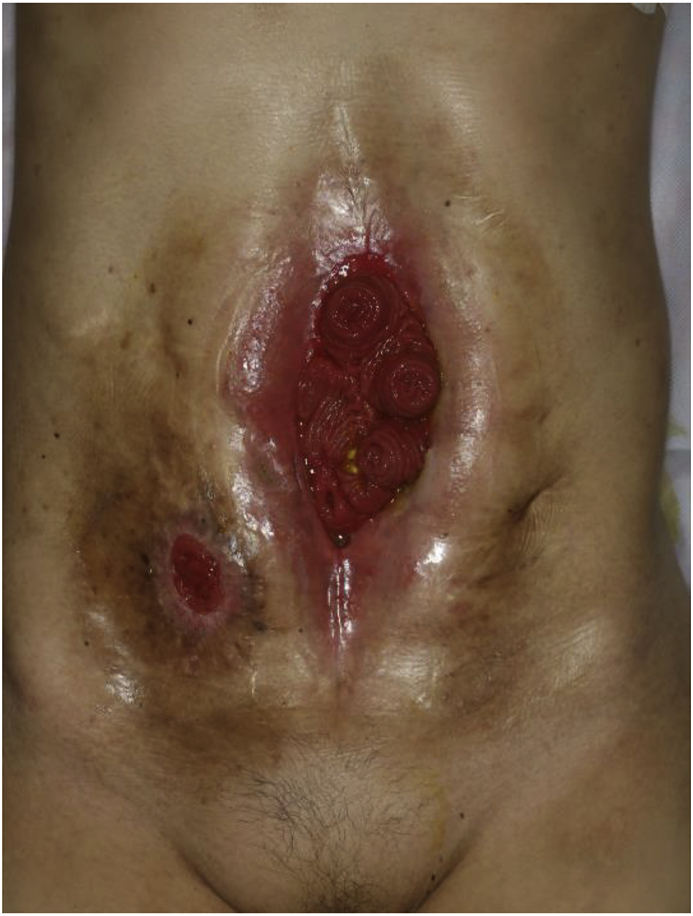
Enterocutaneous cutaneous fistula was widespread, from the xiphoid to the pubis (22 × 5 cm).

**Fig. 2:**
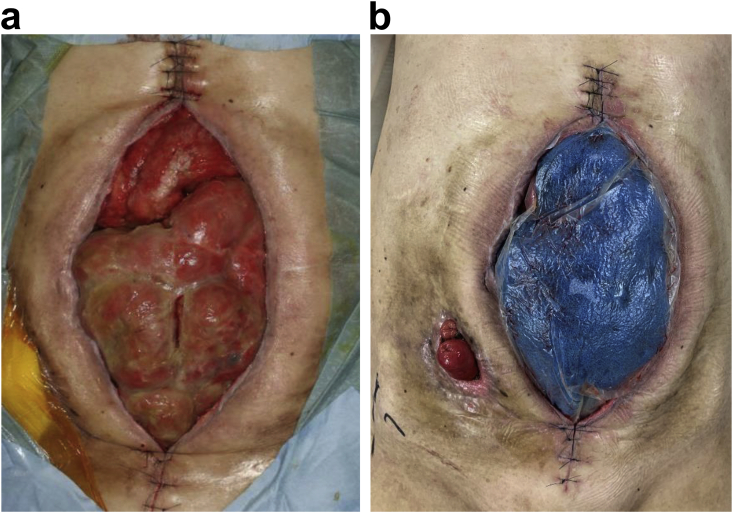
Extent of the abdominal wall defect due to enterocutaneous cutaneous fistula. a) Adding fascia sutures: the abdominal wall defect area was reduced to 18 × 8 cm. b) Continued NPWT for adding fascia sutures.

**Fig. 3:**
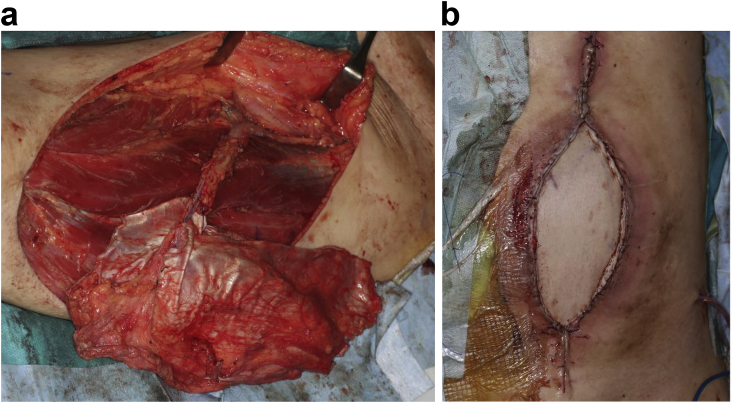
Two-stage abdominal wall reconstruction. a) A left thigh pedicled muscle cutaneous flap was created. b) Abdominal wall reconstruction was completed without complications.

**Fig. 4:**
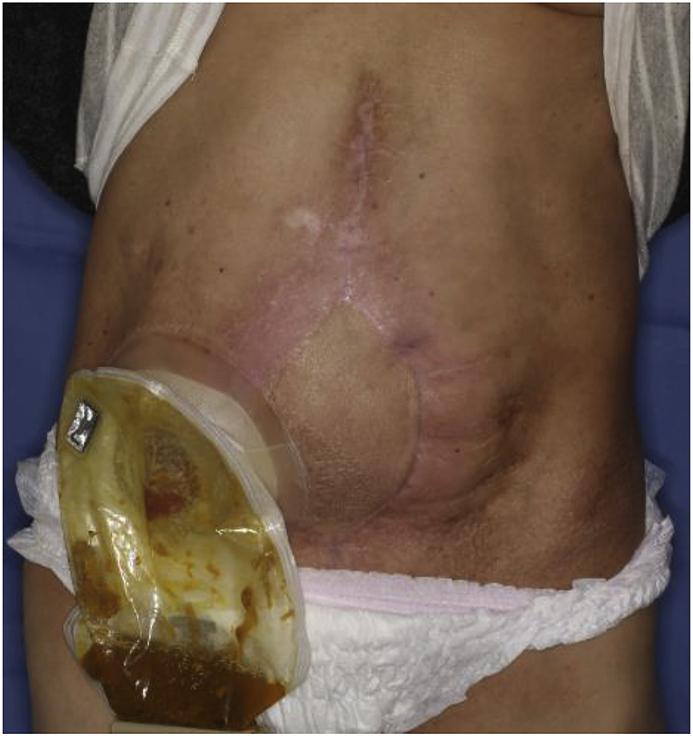
The condition of the postoperative wound. The patient was free of recurrence on postoperative day 90.

A preoperative gastrografin enema image showed six intestinal stumps of EAF, which revealed that the small-intestinal tract had complexly adhered. The extent of the abdominal wall defect due to EAF was widespread, from the xiphoid to the pubis (22 × 5 cm) (Fig. 1). A large amount of intestinal fluid leaked into the abdominal cavity during detachment of the adhesions, and it was expected that intraperitoneal contamination would be high. Thus, detachment of the adhesions, small intestine anastomosis, and ileostomy were performed first. After the initial surgery, intraperitoneal infection control was performed using OAM for a two-stage abdominal wall reconstruction. We used the ABThera™ Open Abdomen Negative Pressure Therapy System (Kinetic Concepts Inc) to change dressings every few days during OAM. Negative pressure was fixed to −125 mmHg each instance. Fascia sutures were added when changing dressings (Fig. 2). Two-stage abdominal wall reconstruction was finally performed 14 days after initial surgery. In spite of the use of fascia sutures, the abdominal wall defect was wide (18 × 8 cm), and therefore, a left thigh pedicled muscle cutaneous flap was created (Fig. 3). Percutaneous drainage of a postoperative abdominal remnant abscess was required, but she was discharged 51 days after the first operation without reoperation. She was recurrence-free on postoperative day 180 (Fig. 4).

This study was conducted in accordance with the Declaration of Helsinki and was reported in line with the SCARE 2018 criteria.^8^ The study conforms to the Pharmaceutical Affairs Law of Japan and was conducted in accordance with Good Clinical Practice guidelines. Written informed consent was obtained from the patient for publication of this case report and accompanying images. A copy of the written consent is available for review by the Editor-in-Chief of this journal on request.

Registration details: unique identifying number (UIN) - researchregistry5571 and reference link - https://www.researchregistry.com/register-now#user-researchregistry/registerresearchdetails/5eb0f5fb31956f0015df6f87/

## 3 Discussion

The mortality rate of ECF ranges from less than 10% to around 20%.^9^^,^^10^ And EAF has no overlying soft tissue and no real fistula tract, making spontaneous healing difficult.^11^ In the present case, in addition to these problems, there were problems of the scarred abdominal wall, skin damage from intestinal fluid exposure for 30 months and intraperitoneal contamination by intraoperative intestinal fluid. And the timing of surgery for EAF was suggested to be delayed for 6–12 months ^12^, but in the present case, it had been 30 months. These problems have been greatly improved thanks to OAM with NPWT same as early intervention cases.

By the use of NPWT, the scarred abdominal wall of long-term EAF 30 months after the onset was softened. NPWT protects the surrounding skin, which assists in dressing adherence for future surgery, and provides the surrounding tissues to help with healing and exudate control.^13^ And NPWT also improves wound healing by increasing blood perfusion and angiogenesis through upregulation of vascular endothelial growth factor and angiopoietin-2.^14^ As a result, abdominal wall reconstruction was performed at intervals of about 1 week after the initial operation.^15^ In the present case, the scarred abdominal wall was stronger than in the early intervention cases, so the waiting period was extended by 2 weeks than usual. And, after 1 week of NPWT, softening of the abdominal wall was observed slowly. So for the rest of 1 week, we performed the addition of fascia sutures to reconstruct the abdominal wall during changes of dressing of NPWT. Pliakos et al. reported that the vacuum-assisted closure technique with the retentions sutured sequential fascial closure had a higher rate of successful abdominal closure than vacuum-assisted closure alone.^16^ In the present case, the size of the abdominal wall defect increased to 25 × 11.5 cm during the initial operation. Moreover, by adding fascia sutures, it was possible to reduce the abdominal wall defect area to 18 × 8 cm. In this manner, the use of NPWT enabled the softening of the scarred abdominal wall of long-term EAF 30 months after the onset, and we performed additional treatment, like fascia sutures, for definitive surgery.

The use of OAM was possible to control infection in the perioperative period. In the present case, the infection in the acute phase at the onset of EAF could be controlled. However, there were six intestinal stumps of EAF and large amounts of small-intestinal fluid from the stumps, ranging from 1500 to 2000 ml/day. The abdominal wall had been exposed to a large amount of small-intestinal fluid for 30 months, and it was expected to leak into the abdominal cavity during detachment of the adhesions. We therefore assumed that perioperative infection would be severe. In fact, because there were more small-intestinal adhesions than expected, the operation time was prolonged, and small-intestinal fluid leakage during the operation was about 800 ml, which required sufficient treatment for peritonitis. OAM by adding NPWT has been reported to be effective in removing residual fluid from the deep intraperitoneal cavity.^17^ During the OAM procedure, effective drainage made it possible to prepare without any complications of intraperitoneal infection, and to be ready for definitive abdominal wall reconstruction.

Recently, there have been an increasing number of reports of OAM with NPWT for traumatic diseases, severe peritonitis, severe pancreatitis and ECF.[18], [19] It is also thought that OAM with NPWT is useful for chronic cases which the timing of surgical intervention was delayed, such as the present case. In the future, the increased spread of indications of OAM for nontraumatic diseases may lead to favorable postoperative outcomes.

## 4 Conclusion

In the present case, we report the successful definitive surgical management for long-term intractable EAF using OAM with NPWT. We believe that a surgical strategy using OAM with NPWT for long-term EAF will have a good postoperative outcome with the same strategy as early intervention of EAF.

## Declaration of competing interest

None.

## Registration of research studies

1. Name of the registry: Kyota Tatsuta
2. Unique identifying number or registration ID: researchregistry5571
3. Hyperlink to your specific registration (must be publicly accessible and will be checked):
https://www.researchregistry.com/register-now#user-researchregistry/registerresearchdetails/5eb0f5fb31956f0015df6f87/

## Availability of data and material

Data sharing is not applicable to this article as no datasets were generated or analyzed during the study.
